# Supplementary material for: A Comparison between Three Tuning Strategies for Gaussian Kernels in the Context of Univariate Genomic Prediction
Source: Genes (Basel). 2022 Dec 3;13(12):2282. doi: 10.3390/genes13122282 (PMC9778581; doi:10.3390/genes13122282)
Supplement: Supplementary file 1 [file genes-13-02282-s001.zip › genes-1985157-supplementary.pdf]

Dataset 4 (Wheat 4)

Prediction performance in terms of MSE

The observed MSE for environment YT_16_17 were 0.106, 0.104 and 0.122 for methods BO, GrS and NT, respectively. For the YT_17_18 environment, the MSE observed were 0.141 (BO), 0.141 (GrS) and 0.165 (NT), while across environments (Global) the observed MSE were 0.114 (BO), 0.113 (GrS) and 0.132 (NT). See **Figure S1A**. For more details, see **Table S1**.

The observed relative efficiencies in terms of MSE for comparison NT/BO were 1.155, 1.166 and 1.159 for environments YT_16_17, YT_17_18 and across environments (Global), respectively. This means that BO had a better prediction performance than NT in every environment by 15.5% (YT_16_17), 16.6% (YT_17_18) and 15.9% (Global). For the comparison between NT/GrS, the observed relative efficiencies were 1.168 (YT_16_17), 1.169 (YT_17_18) and 1.168 (Global). That is, GrS outperformed NT in every environment by 16.8% (YT_16_17), 16.9% (YT_17_18) and 16.8% (Global). Finally, the observed relative efficiencies for the case GrS/BO were 0.989 (YT_16_17), 0.997 (YT_17_18) and 0.992 (Global). This means that both GrS and BO methods had similar performance in every environment (see **Figure S1B**). For more details, see **Table S2**.

Prediction performance in terms of NRMSE

Next, we compared the three strategies of tuning in terms of NRMSE. We observed that for environment YT_16_17 the NRMSE were 0.915, 0.910 and 0.981 for methods BO, GrS and NT, respectively, while for environment YT_17_18 the NRMSE were 0.907 (BO), 0.905 (GrS) and 0.979 (NT), and across environments (Global), the NRMSE were 0.897 (BO), 0.893 (GrS) and 0.964 (NT) (see **Figure S1C**). For more details, see **Table S1.**

The observed relative efficiencies in terms of NRMSE in the comparison NT/BO were 1.072, 1.079 and 1.074 for environments YT_16_17, YT_17_18 and across

environments (Global), respectively. This means that BO outperformed NT in terms of prediction accuracy in every environment by 7.2% (YT_16_17), 7.9% (YT_17_18) and 7.4% (Global). In the comparison NT/GrS the observed relative efficiencies were 1.078 (YT_16_17), 1.082 (YT_17_18) and 1.079 (Global). That is, GrS outperformed NT in every environment by 7.8% (YT_16_17), 8.2% (YT_17_18) and 7.9% (Global). For the comparison of GrS/BO the relative efficiencies were 0.994 (YT_16_17), 0.997 (YT_17_18) and 0.996 (Global). This means that both methods had almost no difference in prediction performance (see **Figure S1D**). For more details, see **Table S2**.

Dataset 5 (Wheat 5)

Prediction performance in terms of MSE

The observed MSE for environment YT_17_18 were 0.078, 0.077 and 0.085 for methods BO, GrS and NT, respectively. For the YT_18_19 environment the MSE observed were 1.81 (BO), 0.180 (GrS) and 0.189 (NT), while across environments (Global), the observed MSE were 0.102 (BO), 0.101 (GrS) and 0.110 (NT) (see **Figure S2**). For more details, see **Table S3**.

The observed relative efficiencies in terms of MSE for comparison NT/BO were 1.101, 1.041 and 1.085 for environments YT_17_18, YT_18_19 and across environments (Global), respectively. This means that BO had a better prediction performance than NT in every environment by 10.1% (YT_16_17), 4.1% (YT_17_18) and 8.5% (Global). For the comparison between NT/GrS, the observed relative efficiencies were 1.108 (YT_17_18), 1.046 (YT_18_19) and 1.093 (Global). That is, GrS outperformed NT in every environment by 10.8% (YT_17_18), 4.6% (YT_18_19) and 9.3% (Global). Finally, the observed relative efficiencies for the case GrS/BO were 0.994 (YT_17_18), 0.995 (YT_18_19) and 0.992 (Global). This means that both GrS and BO methods had similar performance in every environment (see **Figure S2B**). For more details, see **Table S4**.

Prediction performance in terms of NRMSE

Next, we compared the three strategies of tuning in terms of NRMSE. We observed that for environment YT_17_18 the NRMSE were 0.851, 0.849 and 0.893 for methods BO, GrS and NT, respectively, while for environment YT_18_19 the NRMSE were 0.860 (BO), 0.858 (GrS) and 0.878 (NT), and for environment across environments (Global), the NRMSE were 0.841 (BO), 0.838 (GrS) and 0.876 (NT) (see **Figure S2C**). For more details, see **Table S3.**

The observed relative efficiencies in terms of NRMSE in the comparison NT/BO were 1.049, 1.021 and 1.042 for environments YT_17_18, YT_18_19 and across environments (Global), respectively. This means that BO outperformed NT in terms of prediction accuracy in every environment by 4.9% (YT_17_18), 2.1% (YT_18_19) and 4.2% (Global). In the comparison NT/GrS the observed relative efficiencies were 1.052 (YT_17_18), 1.023 (YT_18_19) and 1.045 (Global). That is, GrS outperformed NT in every environment by 5.2% (YT_17_18), 2.3% (YT_18_19) and 4.5% (Global). For the comparison of GrS/BO the relative efficiencies were 0.997 (YT_17_18), 0.998 (YT_18_19) and 0.997 (Global). This means that both methods had almost no difference in prediction performance (see **Figure S2D**). For more details, see **Table S4**.

Dataset 6 (Wheat 6)

Prediction performance in terms of MSE

The observed MSE for environment YT_18_19 were 0.111, 0.112 and 0.117 for methods BO, GrS and NT, respectively. For the YT_19_20 environment the MSE observed were 1.02 (BO), 0.106 (GrS) and 0.113 (NT). While across environments (Global) the observed MSE were 0.109 (BO), 0.111 (GrS) and 0.116 (NT) (see **Figure S3**). For more details, see **Table S5**.

The observed relative efficiencies in terms of MSE for comparison NT/BO were 1.054, 1.108 and 1.064 for environments YT_18_19, YT_19_20 and across environments (Global), respectively. This means that BO had a better prediction performance than NT in every environment by 5.4% (YT_18_19), 10.8% (YT_19_20) and 6.4% (Global). For the comparison between NT/GrS, the observed relative efficiencies were 1.045 (YT_18_19), 1.066 (YT_19_20) and 1.045 (Global). That is, GrS outperformed NT in every environment by 4.5% (YT_18_19), 6.6% (YT_19_20) and 4.5% (Global). Finally, the observed relative efficiencies for the case GrS/BO were 1.009 (YT_17_18), 1.039 (YT_18_19) and 1.018 (Global). This means that both GrS and BO methods had similar performance in every environment (see **Figure S3B**). For more details, see **Table S6**.

Prediction performance in terms of NRMSE

Next, we compared the three strategies of tuning in terms of NRMSE. We observed that for environment YT_18_19 the NRMSE were 0.825, 0.830 and 0.849 for methods BO, GrS and NT, respectively. For environment YT_19_20, the NRMSE were 0.900 (BO), 0.915 (GrS) and 0.940 (NT), while for environment across environments (Global), the NRMSE were 0.465 (BO), 0.468 (GrS) and 0.478 (NT) (see **Figure S3C**). For more details, see **Table S5.**

The observed relative efficiencies in terms of NRMSE in the comparison **NT/BO** were 1.029, 1.044 and 1.028 for environments YT_18_19, YT_19_20 and across environments (Global), respectively. This means that **BO** outperformed **NT** in terms of prediction accuracy in every environment by 2.9% (YT_18_19), 4.4% (YT_19_20) and 2.8% (Global). In the comparison **NT/GrS,** the observed relative efficiencies were 1.023 (YT_18_19), 1.027 (YT_19_20) and 1.021 (Global). That is, **GrS** outperformed **NT** in every environment by 2.3% (YT_18_19), 2.7% (YT_19_20) and 2.1% (Global). For the comparison of **GrS/BO,** the relative efficiencies were 1.006 (YT_18_19), 1.017 (YT_19_20) and 1.006

(Global). This means that both methods had almost no difference in prediction performance (see **Figure S3D**). For more details, see **Table S6.**

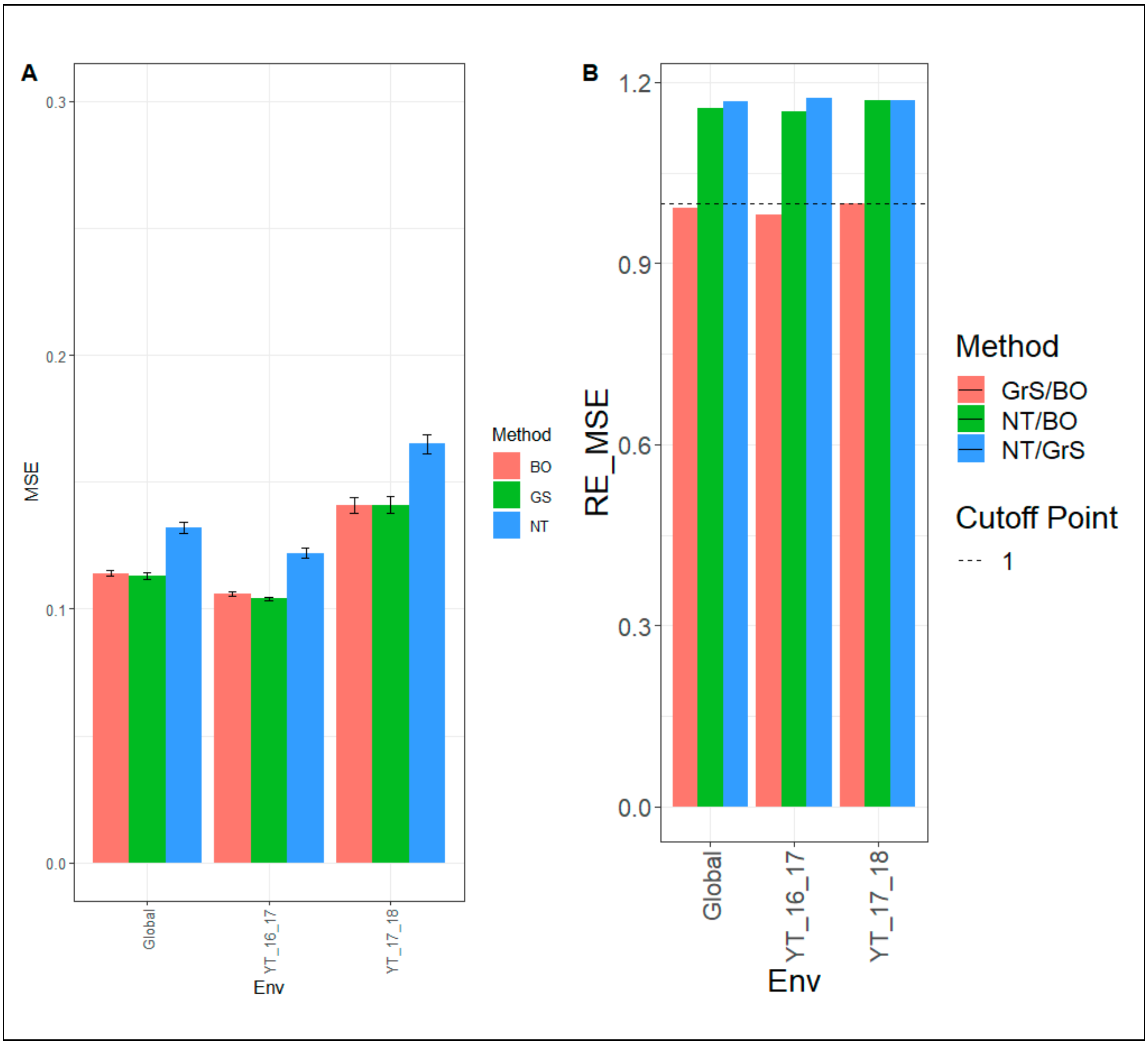

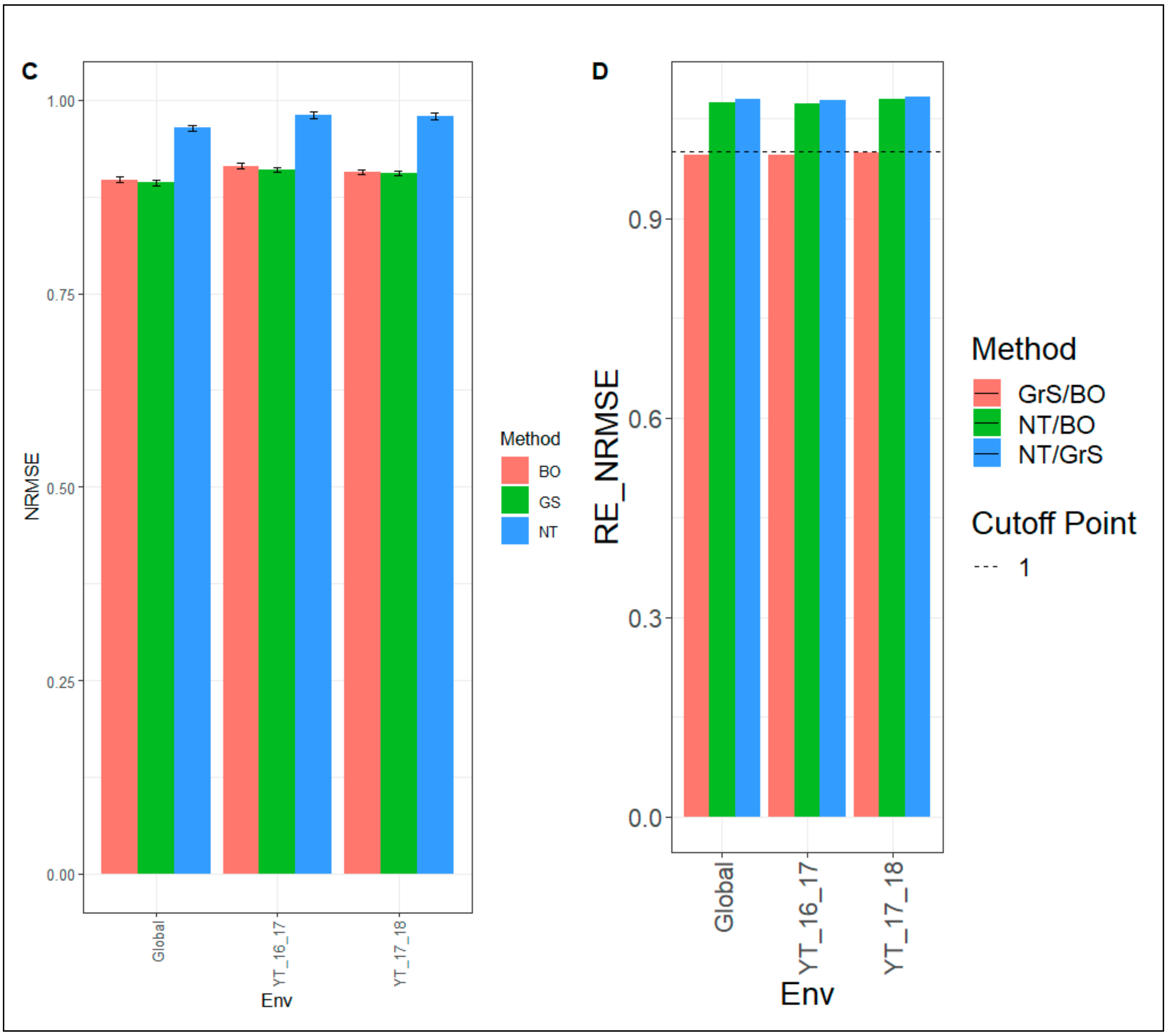

Figure S1. **Dataset 4 (Wheat 4)** A) Mean Square Error (MSE) and their corresponding Standard Error (SE) of the methods Bayesian Optimization (BO), GridSearch Optimization (GrS) and No Tuning (NT) for each environment and across environments of the dataset 4. B) Relative efficiency in terms of the mean squared error (RE_MSE) computed by dividing the MSE of NT and BO; NT and GrS; or GrS and BO. Prediction performance is reported for each environment and across environments in the dataset 4. When RE_MSE>1, the denominator method outperforms the numerator in terms of prediction performance. C) Normalized Root Mean Square Error (NRMSE) and their corresponding Standard Error (SE) of the methods Bayesian Optimization (BO), GridSearch Optimization (GrS) and No Tuning (NT) for each environment and across environments of the dataset 4. D) Relative efficiency in terms of the normalized mean squared error (RE_NRMSE) computed by dividing the NRMSE of NT and BO; NT and GrS; or GrS and BO. Prediction performance is reported for each environment and across

environments in the dataset 4. When RE_NRMSE>1, the denominator method outperforms the numerator in terms of prediction performance.

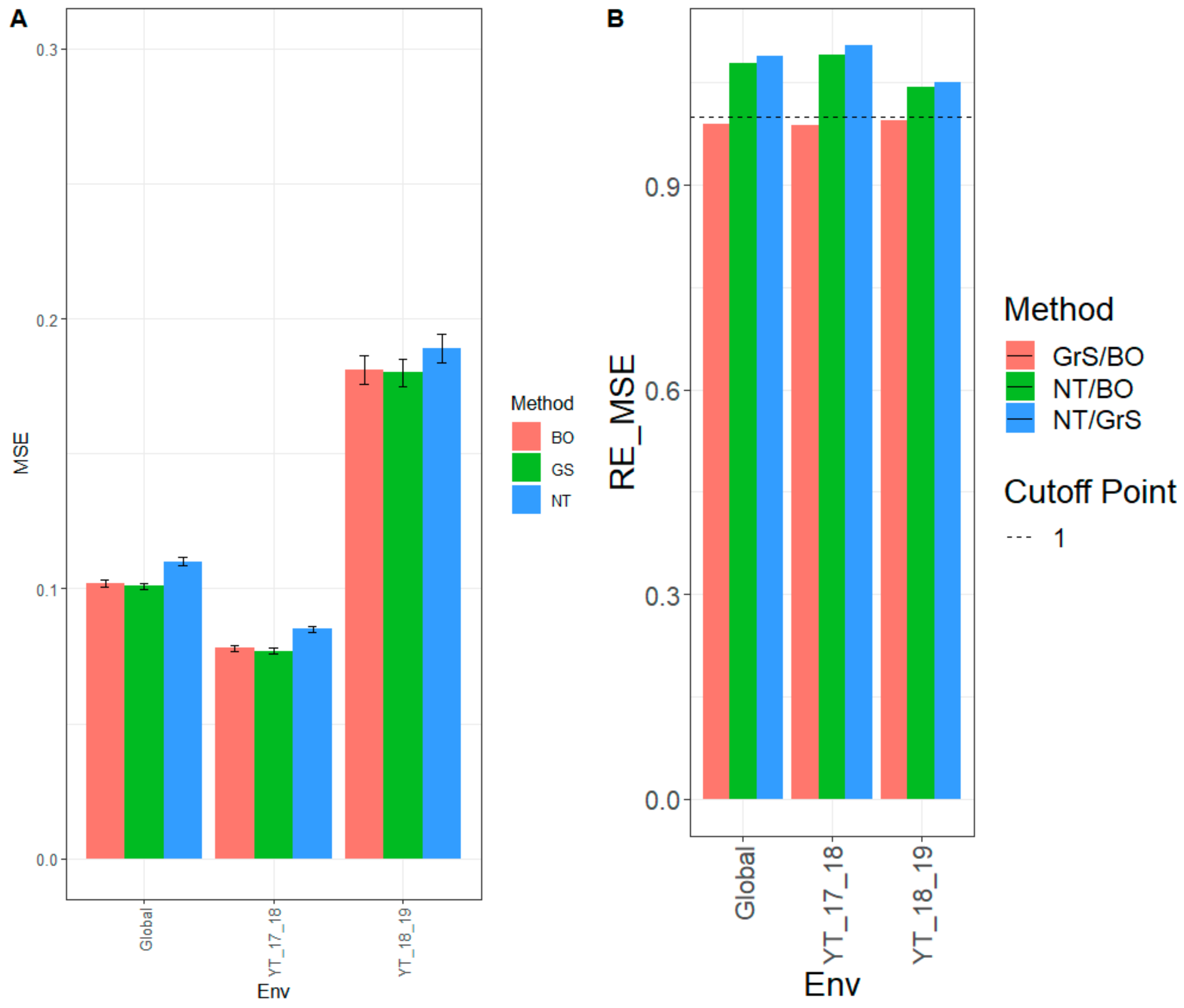

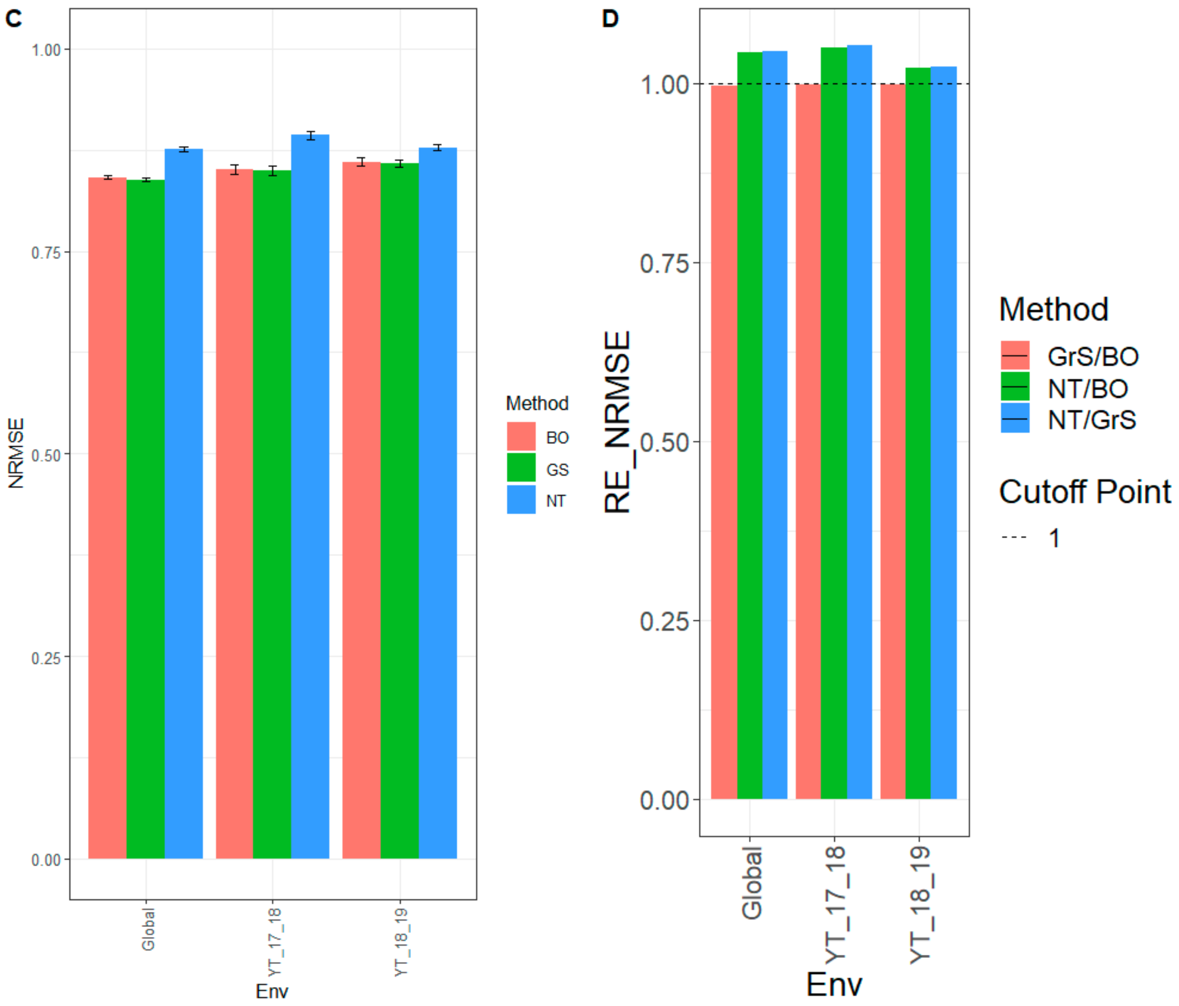

Figure S2. Dataset 5 (Wheat 5). A) Mean Square Error (MSE) and their corresponding Standard Error (SE) of the methods Bayesian Optimization (BO), GridSearch Optimization (GrS) and No Tuning (NT) for each environment and across environments of the dataset 5. B) Relative efficiency in terms of the mean squared error (RE_MSE) computed by dividing the MSE of NT and BO; NT and GrS; or GrS and BO. Prediction performance is reported for each environment and across environments in the dataset 5. When RE_MSE>1, the denominator method outperforms the numerator in terms of prediction performance. C) Normalized Root Mean Square Error (NRMSE) and their corresponding Standard Error (SE) of the methods Bayesian Optimization (BO), GridSearch Optimization (GrS) and No Tuning (NT) for each environment and across environments of the dataset 5. D) Relative efficiency in terms of the normalized mean squared error (RE_NRMSE) computed by dividing the NRMSE of NT and BO; NT and GrS; or GrS and BO. Prediction performance is reported for each environment and across

environments in the dataset 5. When RE_NRMSE>1 the denominator method outperforms the numerator in terms of prediction performance.

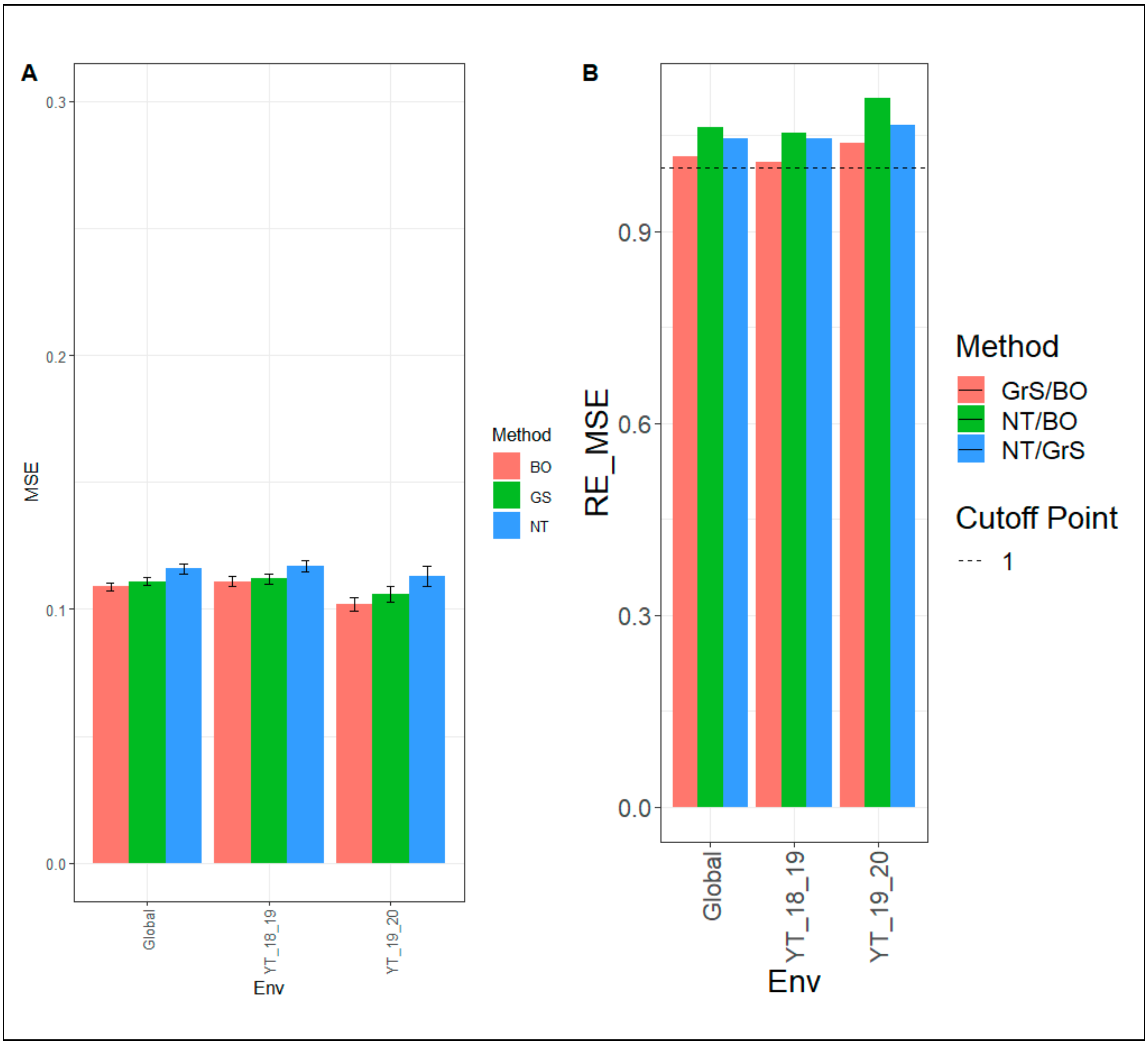

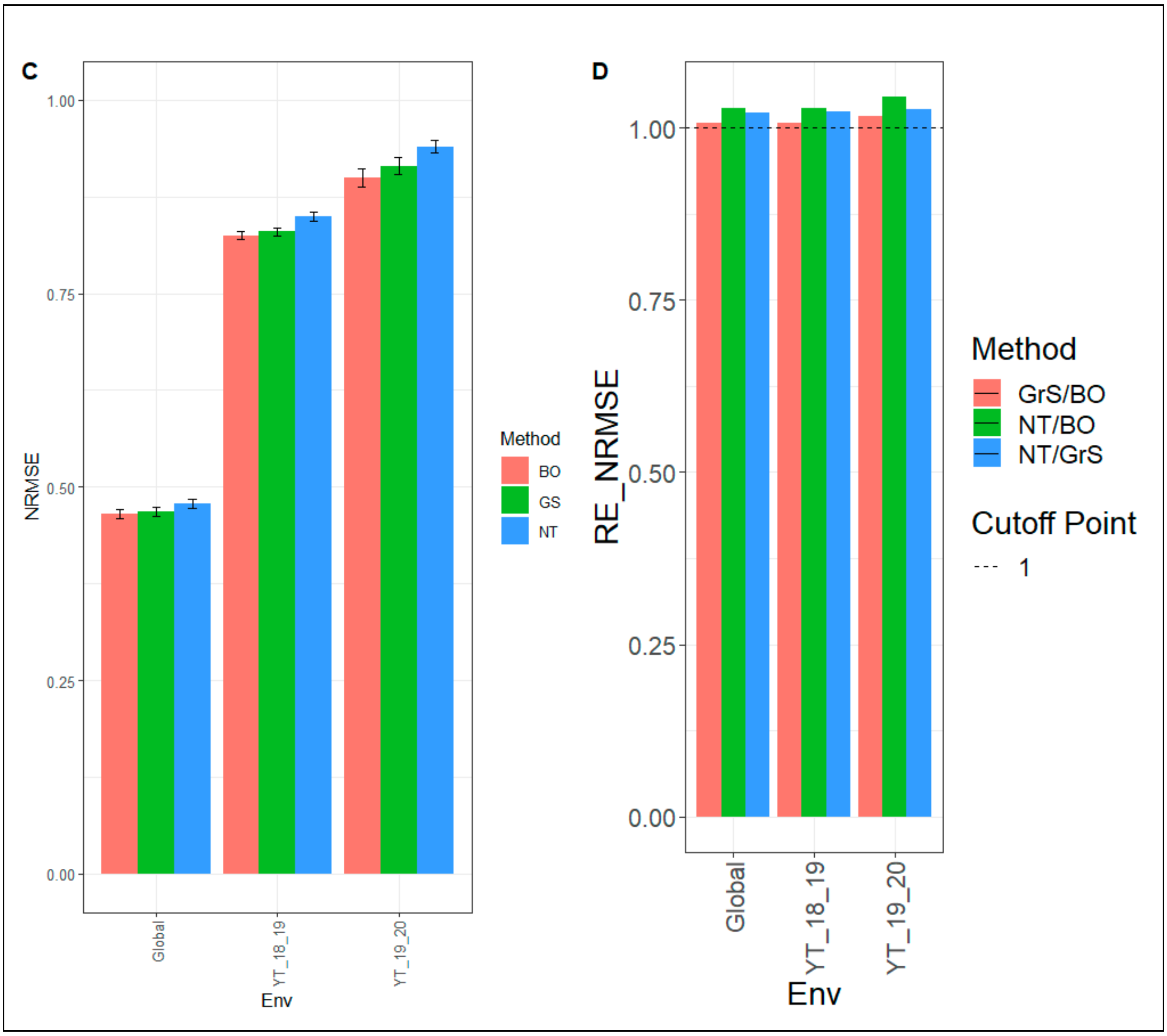

Figure S3. **Dataset 6 (What 6)**. A) Mean Square Error (MSE) and their corresponding Standard Error (SE) of the methods Bayesian Optimization (BO), GridSearch Optimization (GrS) and No Tuning (NT) for each environment and across environments of the dataset 6. B) Relative efficiency in terms of the mean squared error (RE_MSE) computed by dividing the MSE of NT and BO; NT and GrS; or GrS and BO. Prediction performance is reported for each environment and across environments in the dataset 6. When RE_MSE>1 the denominator method outperforms the numerator in terms of prediction performance. C) Normalized Root Mean Square Error (NRMSE) and their corresponding Standard Error (SE) of the methods Bayesian Optimization (BO), GridSearch Optimization (GrS) and No Tuning (NT) for each environment and across environments of the dataset 6. D) Relative efficiency in terms of the normalized mean squared error (RE_NRMSE) computed by dividing the NRMSE of NT and BO; NT and GrS; or GrS and BO. Prediction performance is reported for each environment and across

environments in the dataset 6. When RE_NRMSE>1 the denominator method outperforms the numerator in terms of prediction performance.

Table S1. Prediction performance for every environment and across environments (Global) of the **dataset 4** in terms of their Mean Square Error (MSE), Normalized Root Mean Square Error (NRMSE) under three methods of tuning (BO, GrS and NT). MSE_SE and NRMSE_SE denotes the Standard Errors (SE) under MSE and NRMSE.

Dataset	Method	Env	MSE	MSE_SE	NRMSE	NRMSE_SE
Wheat 4	BO	YT_16_17	0.106	0.002	0.915	0.008
Wheat 4	BO	YT_17_18	0.141	0.008	0.907	0.009
Wheat 4	BO	Global	0.114	0.003	0.897	0.009
Wheat 4	GS	YT_16_17	0.104	0.002	0.91	0.007
Wheat 4	GS	YT_17_18	0.141	0.009	0.905	0.008
Wheat 4	GS	Global	0.113	0.004	0.893	0.008
Wheat 4	NT	YT_16_17	0.122	0.005	0.981	0.011
Wheat 4	NT	YT_17_18	0.165	0.01	0.979	0.013
Wheat 4	NT	Global	0.132	0.006	0.964	0.01

Table S2. Prediction performance for each environment and across environments (Global) of **dataset 4** in terms of their Relative Efficiencies (RE) under two metrics (MSE and NRMSE). In the column method we represent the two tuning strategies that were used to compute their RE_MSE and RE_NRMSE.

Datasets	Env	Method	RE_MSE	RE_NRMSE
Wheat 4	YT_16_17	NT/BO	1.155	1.072
Wheat 4	YT_16_17	NT/GrS	1.168	1.078
Wheat 4	YT_16_17	GrS/BO	0.989	0.994
Wheat 4	YT_17_18	NT/BO	1.166	1.079
Wheat 4	YT_17_18	NT/GrS	1.169	1.082
Wheat 4	YT_17_18	GrS/BO	0.997	0.997

Wheat 4	Global	NT/BO	1.159	1.074
Wheat 4	Global	NT/GrS	1.168	1.079

Table S3. Prediction performance for every environment and across environments (Global) of the **dataset 5** in terms of their Mean Square Error (MSE), Normalized Root Mean Square Error (NRMSE) under three methods of tuning (BO, GrS and NT). MSE_SE and NRMSE_SE denotes the Standard Errors (SE) under MSE and NRMSE.

Dataset	Method	Env	MSE	MSE_SE	NRMSE	NRMSE_SE
Wheat 5	BO	YT_17_18	0.078	0.003	0.851	0.015
Wheat 5	BO	YT_18_19	0.181	0.014	0.86	0.014
Wheat 5	BO	Global	0.102	0.004	0.841	0.006
Wheat 5	GS	YT_17_18	0.077	0.003	0.849	0.015
Wheat 5	GS	YT_18_19	0.18	0.013	0.858	0.012
Wheat 5	GS	Global	0.101	0.003	0.838	0.006
Wheat 5	NT	YT_17_18	0.085	0.003	0.893	0.013
Wheat 5	NT	YT_18_19	0.189	0.014	0.878	0.009
Wheat 5	NT	Global	0.11	0.004	0.876	0.008

Table S4. Prediction performance for each environment and across environments (Global) of **dataset 5** in terms of their Relative Efficiencies (RE) under two metrics (MSE and NRMSE). In the column method we represent the two tuning strategies that were used to compute their RE_MSE and RE_NRMSE.

Datasets	Env	Method	RE_MSE	RE_NRMSE
Wheat 5	YT_17_18	NT/BO	1.101	1.049
Wheat 5	YT_17_18	NT/GrS	1.108	1.052
Wheat 5	YT_17_18	GrS/BO	0.994	0.997
Wheat 5	YT_18_19	NT/BO	1.041	1.021
Wheat 5	YT_18_19	NT/GrS	1.046	1.023

Wheat 5	YT_18_19	GrS/BO	0.995	0.998
Wheat 5	Global	NT/BO	1.085	1.042
Wheat 5	Global	NT/GrS	1.093	1.045

Table S5. Prediction performance for every environment and across environments (Global) of the **dataset 6** in terms of their Mean Square Error (MSE), Normalized Root Mean Square Error (NRMSE) under three methods of tuning (BO, GrS and NT). MSE_SE and NRMSE_SE denotes the Standard Errors (SE) under MSE and NRMSE.

Dataset	Method	Env	MSE	MSE_SE	NRMSE	NRMSE_SE
Wheat 6	BO	YT_18_19	0.111	0.005	0.825	0.013
Wheat 6	BO	YT_19_20	0.102	0.007	0.9	0.031
Wheat 6	BO	Global	0.109	0.004	0.465	0.015
Wheat 6	GS	YT_18_19	0.112	0.005	0.83	0.014
Wheat 6	GS	YT_19_20	0.106	0.008	0.915	0.03
Wheat 6	GS	Global	0.111	0.004	0.468	0.015
Wheat 6	NT	YT_18_19	0.117	0.006	0.849	0.016
Wheat 6	NT	YT_19_20	0.113	0.01	0.94	0.02
Wheat 6	NT	Global	0.116	0.005	0.478	0.016

Table S6. Prediction performance for each environment and across environments (Global) of **dataset 6** in terms of their Relative Efficiencies (RE) under two metrics (MSE and NRMSE). In the column method we represent the two tuning strategies that were used to compute their RE_MSE and RE_NRMSE.

Datasets	Env	Method	RE_MSE	RE_NRMSE
Wheat 6	YT_18_19	NT/BO	1.054	1.029
Wheat 6	YT_18_19	NT/GrS	1.045	1.023
Wheat 6	YT_18_19	GrS/BO	1.009	1.006

Wheat 6	YT_19_20	NT/BO	1.108	1.044
Wheat 6	YT_19_20	NT/GrS	1.066	1.027
Wheat 6	YT_19_20	GrS/BO	1.039	1.017
Wheat 6	Global	NT/BO	1.064	1.028
Wheat 6	Global	NT/GrS	1.045	1.021
Wheat 6	Global	GrS/BO	1.018	1.006
